# Defining the complex needs of families with rare diseases—the example of telomere biology disorders

**DOI:** 10.1038/s41431-024-01697-6

**Published:** 2024-10-01

**Authors:** Catherine Wilsnack, Camella J. Rising, Emily E. Pearce, Rowan Forbes Shepherd, Ashley S. Thompson, Alina Majid, Allison Werner-Lin, Sharon A. Savage, Sadie P. Hutson

**Affiliations:** 1 Steve Hicks School of Social Work, University of Texas at Austin, Austin, TX USA; 2 Clinical Genetics Branch, Division of Cancer Epidemiology and Genetics, National Cancer Institute, Rockville, MD USA; 3 Health Care Delivery Research Program, Office of the Associate Director, Division of Cancer Control and Population Sciences, National Cancer Institute, Rockville, MD USA; 4 School of Social Policy and Practice, University of Pennsylvania, Philadelphia, PA USA

**Keywords:** Quality of life, Social sciences, Genetics

## Abstract

Families with rare diseases, such as telomere biology disorders (TBDs), may have extensive unmet needs given the heterogeneity, chronicity, and potential severity of illness. TBDs are rare inherited syndromes associated with high risk of bone marrow failure, cancer, pulmonary fibrosis, and other severe, chronic complications. To identify gaps in clinical care, we aimed to ascertain the perceived unmet needs of adults and family caregivers, current or bereaved, of individuals with TBDs. Participants were aged ≥18 years with a self-reported TBD diagnosis and/or ever caregivers to one or more family members with a TBD. Participants completed an online survey (*N* = 35) and/or an audio-recorded telephone interview (*N* = 32). We calculated descriptive statistics in SPSS and thematically analyzed interview transcripts. Quantitative and qualitative data were analyzed concurrently. Most participants were aged ≥35 years, female, highly educated, and medically insured. Survey respondents reported numerous unmet needs in psychosocial, medical, financial, and daily activity domains. In interviews, participant descriptions validated and contextualized the salience of these unmet needs. Both qualitative and quantitative data identified critical shortfalls in addressing chronic family distress and specialty care coordination. Adults and caregivers of individuals with TBDs have a high risk of adverse psychosocial sequelae given extensive unmet needs. These findings provide a foundation for understanding the range and extent of gaps in care for families with rare diseases, especially TBDs but that are likely applicable to others. Tailored multi-disciplinary interventions involving patients, families, clinicians, researchers, and patient advocacy communities are required to appropriately address care needs for all rare diseases.

## Introduction

Estimates suggest there are at least 10,000 rare diseases affecting at least 30 million people in the United States (US) [1, 2]. In 2019, the estimated financial burden of just 379 rare diseases was estimated at $997 billion US dollars [3]. Individuals living with rare disease often experience a spectrum of illness-related symptoms that are not well understood, which significantly prolongs time to diagnosis and limits access to disease-appropriate, patient-centered care [4, 5]. Patients and their caregivers experience chronic medical uncertainty due in part to the complex clinical manifestations, difficulties accessing expert medical care, limited therapeutic modalities, and challenges navigating medical care systems [6, 7]. These challenges often result in extensive needs in multiple life domains [6–8]. Empirical studies of certain rare disease populations have uncovered illness-specific issues, healthcare barriers, psychosocial risks, financial burdens, and other complex and dynamic challenges [8, 9].

Telomere biology disorders (TBDs) are a group of inherited conditions caused by rare pathogenic germline variants in genes responsible for regulating telomere maintenance [10–13]. At least 17 different genes are associated with TBDs and can be inherited in X-linked recessive, autosomal dominant, or autosomal recessive patterns, resulting in a wide spectrum of phenotypes [14]. Dyskeratosis congenita (DC), the prototypic TBD, is classically diagnosed by the triad of abnormal skin pigmentation, oral leukoplakia, and dysplastic nails. Hoyeraal-Hreidarsson syndrome presents in early childhood with features of DC plus cerebellar hypoplasia and immunodeficiency. Patients with TBDs are at high risk of bone marrow failure, cancer, liver disease, pulmonary fibrosis, and other complications. In addition to variable expressivity, TBDs have variable levels of penetrance and genetic anticipation across affected generations, resulting in different phenotypes, even within families [10, 11, 14]. Therapeutic options are limited to hematopoietic and/or solid organ transplant, and supportive care.

Like many other rare diseases, the medical and psychosocial needs of individuals with TBDs and their caregivers are likely extensive [15, 16]. For example, individuals with Fanconi anemia (FA), a rare cancer-prone inherited bone marrow failure syndrome with similar features to TBDs, report challenges related to diagnoses, treatment decision-making, seeking and comprehending genetic illness information, and managing distress [17–19]. Parents of children with FA also reported chronic uncertainty, distress, and lack of support from healthcare systems and providers [20]. This study aimed to ascertain the perceived unmet needs of adults and current or bereaved family caregivers of individuals with TBDs and identify gaps in clinical care.

## Materials and methods

### Study participants

This study was approved by the National Institutes of Health (NIH) IRB (NIH Protocol 000502-C, ClinicalTrials.gov, Identifier NCT04959188). We recruited individuals through Team Telomere, Inc. communications, including email and social media, and at an in-person camp for families with TBDs. Team Telomere is a non-profit TBD advocacy organization (https://teamtelomere.org/). We also recruited participants enrolled in the National Cancer Institute (NCI) inherited bone marrow failure syndrome study (NIH Protocol 02-C-0052, ClinicalTrials.gov, Identifier NCT00027274) via email or at clinical visits. Eligible individuals were aged ≥18 years and had a self-reported diagnosis of a TBD and/or were ever a caregiver of a family member with a TBD (e.g., spouse, child). Supplementary Material 1 describes clinical manifestations of TBDs [14].

### Design

Our study team employed a concurrent mixed methods design for the needs assessment, with qualitative findings used to validate quantitative results [21]. Our qualitative approach was informed by interpretive description, with an interpretivist framing [22]. Quantitative and qualitative results from the needs assessment are presented separately, and data were mixed at the level of interpretation to clarify convergence and divergence between datasets. Drawing on principles of participatory research design [23], we collaborated with Team Telomere to identify TBD needs assessment topics.

### Data collection

#### Quantitative survey

Eligible participants completed an anonymous online survey using Qualtrics between September 2021 and June 2023. After providing consent, participants responded to demographic items and TBD-related needs in four domains: psychosocial, medical, financial, and daily activity. To assess unmet needs of adults with TBDs and caregivers of a living family member with a TBD, respectively, we used slightly modified versions of the Needs Assessment of Family Caregivers-Cancer (NAFC-C) [24]. To assess unmet needs of bereaved caregivers, we used a slightly modified version of the Needs Assessment of Family Caregivers-Bereaved to Cancer (NAFC-BvC) [25]. The NAFC-C and NAFC-BvC were developed based on Need Fulfillment Theory [24–26]. Prior to making modifications to these instruments, CW consulted with lead author/developer, Dr. Youngmee Kim [24, 25]. Item modifications included changing “cancer” to “illness” and changing the pronouns used on the survey version for adults with TBDs (e.g., changed “his/her cancer” to “you/your illness”). Participants were asked to complete the needs assessment from a single self-selected perspective, an adult with a TBD, a caregiver of a living family member with a TBD, or a bereaved caregiver of a deceased family member with a TBD (see Supplementary Material 2).

After selecting the perspective from which they would complete the assessment, participants indicated needs they experienced in the past four weeks from a listing of 21 to 27 options. Participants rated the importance of each need (0=*not important at all*, 2=*somewhat important*, 4=*extremely important*) and satisfaction with fulfilling that need (0=*not satisfied at all*, 2=*somewhat satisfied*, 4=*extremely satisfied*). At the end of the survey, participants indicated willingness to participate in an interview (*yes*/*no*).

#### Qualitative interviews

We developed a semi-structured interview guide to explore survey domains and understand TBD-related healthcare experiences and impact on daily life. Telephone interviews were audio recorded following participant consent. Interviews were conducted by qualitatively trained pre- and post-doctoral NCI fellows (CW, CJR, EEP, RFS, AST) between September 2021 and July 2023. We transcribed interviews verbatim and anonymized transcripts before analysis. Qualitative interview and survey data cannot be directly linked as surveys were completed anonymously. Survey and interview data were stored on password-protected NIH computers.

### Data Analysis

#### Statistical analysis

We used IBM SPSS Statistics (2021) to calculate descriptive statistics. Needs assessment scores were calculated according to the procedure of Kim and colleagues [24]. Satisfaction scores were reverse coded for each item, with higher scores indicating less satisfaction with fulfilling that need. Needs reported as *not important at all* and/or *extremely satisfied* received a score of zero. Each need item was scored to create an index of unfulfillment by multiplying the importance rating by the reverse coded satisfaction rating. Scores could range from 0 to 16. *Unmet need* was operationalized as any score greater than zero, with higher (versus lower) scores signifying relatively greater unfulfillment of a need. Total mean scores for each of the four domains (psychosocial, medical, financial, and daily activity) were calculated by averaging respective item scores with values greater than zero within each domain, as described by Kim and colleagues [24]. We applied this same procedure to calculate domain scores for the bereaved caregiver group to enable comparison of total mean scores across all three groups.

#### Thematic analysis

We used codebook thematic analysis to develop findings [27]. Coders (CW, CJR, EEP, RFS, AST, AM, SPH) used a hybrid inductive-deductive coding strategy to develop a broad and structured (coded) dataset suitable for different research questions. Our large coding team iteratively developed a working codebook over an extended 6-month period of data familiarization. As a team, and in pairs per codebook section, we discussed and established codebook domains, coding definitions, and parameters for use, leveraging our interprofessional expertise to increase rigor and interpretive richness. Coders analyzed data by assigning conceptual codes to text in Dedoose (Version 9.0.107).

This mixed-method analysis primarily made use of a priori (deductive) codes developed from domains of the needs assessment survey, enabling us to triangulate data types and explore TBD-related needs in greater depth [28]. Themes presented here are semantic or descriptive; they cohere around a shared topic, summarizing key points expressed by participants in relation to a specific need. The purpose was to enrich quantitative needs assessment data with participant voices and describe in more depth the challenges of living with a TBD as they relate theoretically to caregiving needs based on Need Fulfillment Theory [26], as described by Kim and colleagues [24, 25]. Planned future analyses will explore latent meanings across aspects of TBD experiences.

Themes were developed by summarizing deductive (and relevant inductive) codes related to specific needs assessment domains or items (e.g., domain: psychosocial needs; item: coping with emotional distress). Analytic rigor was promoted through discussion of themes from interprofessional perspectives, practicing reflexivity (see Supplementary Material 3), and verifying final themes with experts in qualitative and mixed methods research. The latter involved reviewing candidate themes for dimensionality, internal consistency, relationship to needs assessment topic, and overall fit with the analytical story. Preliminary results were discussed with Team Telomere to ensure confirmability of the findings.

## Results

### Study participants

#### Quantitative survey

Forty participants completed the online survey (Table 1). Survey participants were predominantly female (*n* = 37/40, 93%) and aged 35 years or older (*n* = 34/39, 87%). The majority were college graduates (*n* = 30/39, 77%) and medically insured (*n* = 35/39, 90%). Just over half (*n* = 20/39, 51%) reported an annual household income greater than U.S. $100,000.

**Table 1:** Participant Characteristics.

Characteristic	Survey^a^ (*N* = 40) *n* (%)	Interview^a^ (*N* = 32) *n* (%)
Completed needs assessment from perspective of:		
Adult with a TBD	19 (47.5)^b^	11 (34.4)^c^
Family caregiver	16 (40.0)^b^	9 (28.1)^d^
Family caregiver with a TBD	NA^b^	5 (15.6) ^e^
Bereaved family caregiver	5 (12.5)^b, f^	7 (21.9) ^f^
Age (years)^g^		
25–34	5 (12.8)	4 (12.5)
35–44	13 (33.3)	7 (21.9)
45–54	10 (25.7)	11 (34.4)
≥55	11 (28.2)	10 (31.2)
Gender		
Male	3 (7.5)	4 (12.0)
Female	37 (92.5)	28 (88.0)
Educational attainment^g, h^		
High school graduate or post-high school education	9 (23.1)	NA
College graduate	30 (76.9)	NA
Medical insurance status^g^		
Insured	35 (89.8)	NA
Uninsured	2 (5.1)	NA
Do not know	2 (5.1)	NA
Annual household income (US dollars) ^g^		
<100,000	17 (43.6)	NA
≥100,000	20 (51.3)	NA
Do not know	2 (5.1)	NA

NA indicates not ascertained.

^a^Samples are not independent; participants can be counted multiple times across data collection methods.

^b^Survey respondents included in the analytic sample could only complete the needs assessment from one perspective, as an adult with a TBD, as a family caregiver of a living individual with a TBD, or as a bereaved family caregiver of a deceased individual with a TBD.

^c^Several (*n* = 6) were bereaved of at least one first-degree relative who had a TBD.

^d^All were caregivers to one or more living children with a TBD and one was also a caregiver to a spouse.

^e^All were caregivers to one or more living children with a TBD.

^f^Former caregivers to a spouse (*n* = 4), first-degree relative (*n* = 2), or spouse and first-degree relative (*n* = 1) who had a TBD.

^g^The denominator is 39, not 40, due to 1 missing value in the survey data.

^h^Post-high school education includes some college or technical or vocational school. College graduate includes graduates of an associate, bachelor’s, or post-baccalaureate graduate degree program.

Among those who self-reported a TBD diagnosis (*n* = 22/40, 55%), about one third (*n* = 7/22, 32%) were also current caregivers of at least one family member with a TBD, and nearly three quarters (*n* = 16/22, 73%) were bereaved of at least one deceased family member with a TBD. Those who self-reported no personal TBD diagnosis (*n* = 18/40, 45%) indicated they were current caregivers to at least one family member with a TBD (*n* = 13/18, 72%) and/or bereaved of at least one deceased family member with a TBD (*n* = 8/18, 28%). Several (*n* = 5/18, 28%) identified as bereaved caregivers of a spouse/partner (*n* = 3/5, 60%) or child (*n* = 2/5, 40%).

DC was the most frequently reported TBD-related diagnosis among affected adult survey participants (*n* = 17/23, 74%) and survey participants’ children with TBDs (*n* = 12/16, 75%). Other self-reported diagnoses included pulmonary fibrosis, pulmonary arteriovenous malformations, liver disease, aplastic anemia, cancer, structural brain changes, and Hoyeraal-Hreidarsson syndrome. Over half of survey participants with TBDs (*n* = 12/22, 55%) reported more than one TBD-related diagnosis.

#### Qualitative interviews

Thirty-two participants completed telephone interviews averaging 90 min (range = 59–134 min). Most interview participants were female (*n* = 28/32, 88%), 45 years of age or older (*n* = 21/32, 66%), and resided in the U.S. (*n* = 30/32, 94%). Interview participants reported a personal TBD diagnosis (*n* = 11/32, 34%), currently caregiving to one or more affected family members (*n* = 9/32, 28%), having both a personal TBD diagnosis and currently caregiving (*n* = 5/32, 16%), or formerly caregiving to one or more affected family members (i.e., bereaved) (*n* = 7/32, 22%). Among all 14 caregivers of living affected family members, caregiving was for at least one child with a TBD (ranging from young to adult children), with one also caregiving for a spouse with a TBD. Most interview participants (*n* = 19/32, 59%) were bereaved of at least one deceased family member with a TBD. Those who participated solely as former caregivers were bereaved of a spouse (*n* = 4/7, 57%), child (*n* = 1/7, 14%), or parent and/or sibling (*n* = 2/7, 29%). Thematic analysis of interview data generated four themes corresponding to our needs assessment domains: psychosocial, medical, financial, and daily activity needs. Illustrative quotes are provided in text and Table 2. In Table 2, participant relationship to TBDs and age are provided in parentheses for context.

**Table 2:** Illustrative quotes from adults and family caregivers of individuals with telomere biology disorders (*N* = 32).

Domain/Theme	Direct quote
**Psychosocial domain**	
Dealing with your emotional distress	“It almost feels like I can’t remember a lot of [my bone marrow transplant] just because there is so much trauma in the last year and a half…When I was first diagnosed, there was so much unknown and just so much anxiety about all the different possibilities of things.” (adult with a TBD, age 34)
Helping them with their emotional distress	“He [son with TBD] said, I’m too scared to be angry…And I remember telling him, “I’m so angry that this happened to you buddy.” And he cried for like an hour…I just couldn’t believe that he had so much grief. But I could understand…” (caregiver, age 54)
Talking to anyone about your concerns	“I wish my family was less emotional about it [participant’s TBD] …It’s not like I avoid telling them stuff…But I usually package it well…because if they’re stressed, it adds to my own stress.” (adult with a TBD)
Talking to them about their concerns	“After his [lung] transplant…I was trying to help him [spouse with a TBD] work through this pending doom in the future, and he didn’t like that at all…Anything to do with death was just really difficult for him.” (bereaved caregiver, age 72)
Finding meaning in your/their illness	“I read about the stories [on TBD social media] because I want to find maybe some common aspects of what we are experiencing…The stories are all quite similar with these families that had to deal…with this condition nobody expected…find courage to fight with this, and then to find the positive sides of the conditions…From that point of view, all our stories are quite similar.” (caregiver, age 48)
Being satisfied in the caregiver-receiver relationship	My stress comes from [my son with a TBD] and trying to help him through this and giving him suggestions on what to do, and…nothing is sticking. He’s just like, ‘That won’t help. That won’t help.’ That kind of stuff I think is where my biggest stress comes from.” (caregiver, age 56)
Having an intimate relationship	“This idea of building a future, like romantic relationship or partner, and having to introduce this idea of, like, I want you to love every part of me, but this is a part of me that I don’t love [participant’s TBD]. I don’t know how to introduce that [to a potential partner].” (adult with a TBD, age 27)
Dealing with lifestyle changes	“Before [participant’s spouse’s (with a TBD) lung] transplant, it was like, “Where are we going to live?” because we had to live over by [the hospital]…Is it going to be clean? What about the air filter system? I don’t know how this works with all the things he’s going to need to eat. And who’s going to watch my daughter while I’m over there?” (caregiver, age 57)
**Medical domain**	
Getting the best possible care for yourself/them	“It has been my recent experience in seeking out a lung transplant that there is a reluctance for many health care centers to take on complicated cases…I have been rejected at 4 big centers…For me this has been a huge stress…” (adult with a TBD, age 52)
Communicating with medical staff	“…I feel like a guinea pig. There’s lots of people [health care providers] who have never heard of the disease…And they do a bunch of research, and then they want to ask an hour’s worth of questions when I go in there…I don’t need that.” (adult with a TBD, age 35)
Getting involved in medical decision-making	“When medical decisions have to be made, I think that’s where the uncertainty becomes more of an issue…If I come to a point where I have to have a lung transplant…what’s on the other side of the lung transplant for DC patients [versus for non-DC patients]?” (caregiver with a TBD, age 58)
Getting information about illness	“Even information now, if we had it four years ago, it would have changed our plan for [our son with a TBD]. We wouldn’t have been looking at a double lung transplant…We would have been looking at a liver transplant…I wish we had known…” (caregiver with a TBD, age 46)
Understanding/navigating insurance coverage	“I can’t get insurance on my own because of [my TBD] …I guarantee you with a diagnosis of [a pathogenic germline variant in] *DKC1*, I already know disability is not going to give me anything. It’s going to be one of those that I’ll never get no matter how hard I try…” (adult with a TBD, age 46)
Managing your/their illness-related pain	“It’s like getting yourself to where you can let go of the emotional attachment to the pain [due to post-transplant graft-versus-host disease] and kind of walk through it and keep yourself moving…I just have to get myself in the right mindset through it.” (adult with a TBD, age 49)
Managing your/their other symptoms (non-pain)	“I can only function to an extent on a daily basis. I require a lot of sleep, more sleep than a regular adult female my age would need. By the evening time, I’m tired. I don’t want to do anything…I’m just, I’m out of energy…So, this is the reason why I’m probably not very active in life.” (adult with a TBD, age 32)
**Financial domain**	
Taking care of bills/Having enough insurance coverage	“We are constantly preparing in a different way…My husband, he’s working more. I’m working more… [Our son with a TBD] does his bone marrow [biopsy] every year…And we have a copay…it’s still a lot. 20 percent of a lot is still a lot. We’re kind of building up finances to pay for the things that are going to be required of that.” (caregiver, age 27)
Paying for your/their medical expenses	“I see doctors [regularly] for maintenance and [TBD] side effects…Insurance is really expensive. I make $11/hour…and I’m trying to go to school. I literally can’t pay my bills. Maybe I need to take a year off of medical care.” (adult with a TBD, age 44)
**Daily activity domain**	
Meeting your personal needs	“I’m always still balancing preparing for something and actually living my life…One of the things that I stopped doing when [my son with a TBD] got sick is…I stopped running because he couldn’t run…And so, I just couldn’t enjoy running anymore. I couldn’t enjoy my own sort of physical health.” (caregiver, age 54)
Getting help from others to take time for self	“I think my mom has helped a lot…I’ll just bring the boys [one with a TBD] over there and I can go grocery shopping, like, at the actual store and not have to worry about exposing them [to viruses, etc.], and just walking around the store and looking at things and just kind of, you know, not having to worry about anything.” (caregiver, age 41)
Taking time off work	“The only time I’ve taken off time for work was during [my son’s bone marrow] transplant, and even that was really hard to manage. I ended up having to resign from my job…We didn’t know if he was going to die, and we wanted to spend as much time together as we could.” (caregiver, age 44)
Getting together with family and friends	“I can’t be around a lot of people for anything [due to COVID-19]…I don’t go in restaurants to eat. I only eat outside with friends. I don’t gather with any large group of anybody. Even my grandkids, I’m only around them with masks on because they’re in school and they’re too young to be vaccinated. So, they’re a major risk to me.” (caregiver with a TBD, age 61)
Being satisfied with family/friend relationships	“When my mom got sick and she passed, it was so traumatic to my family…For me to turn back around however many years later and tell them, ‘Okay, I have the same diagnosis’…I didn’t have the most support from them specifically because they were in panic mode of ‘this is all going to happen over again.’” (adult with a TBD, age 49)
Balancing work or school with managing illness	“I had one school counselor…when she [daughter with a TBD] was first diagnosed, and he worked with special needs kids quite a bit…We kept trying to adjust her [school] schedule, and she was fatigued, and she had really bad headaches, and [we were] trying to keep her life normal.” (caregiver with a TBD, age 51)
Reorganizing roles in family	“It’s big to kind of hand over. For me as a parent, I can’t be like [to young adult son], ‘Oh, did you go to the ENT this month?’ or ‘Did you go see the GI doctor? Can I still come with you?’ Like, how does this work?” (caregiver, age 44)
Finding assistance with your/their daily needs	“I would yell to her [daughter] in the middle of the night…’I need you to come help me move your dad [spouse with a TBD]…’ I would need to change the chucks underneath him at the very end…I couldn’t get him off of the bed onto the commode.” (bereaved caregiver, age 49)

### Needs assessment

Among survey respondents, 19 chose to complete the needs assessment from the perspective of an adult with a TBD and 16 completed it as a caregiver of a living family member with a TBD. Five completed the needs assessment as a bereaved caregiver of a deceased family member with a TBD. In the sections that follow, we describe reported unmet needs of these groups by domain, using both survey and interview data.

#### Psychosocial needs

All adults with a TBD (*n* = 19/19, 100%) and most current (*n* = 14/16, 88%) and bereaved (*n* = 4/5, 80%) family caregivers of individuals with TBDs reported at least one unmet psychosocial need (Table 3). For adults with TBDs and current caregivers, unmet needs related primarily to “dealing with your emotional distress” (adults: *n* = 16/19, 84%; caregivers: *n* = 11/16, 69%) and “helping them with their distress” (caregivers: *n* = 12/16, 75%). More than half of current caregivers (*n* = 10/16, 63%) reported unmet needs regarding “talking to them about their concerns.” Although less frequent, mean scores for “finding meaning in your/their illness” and “having an intimate relationship” were similar, or exceeded, mean scores for dealing with personal or family members’ distress. Other unmet needs reported by both groups included “talking with anyone about your concerns,” “being satisfied in the caregiver-care receiver relationship,” and “dealing with lifestyle changes.” Among bereaved caregivers, more than half reported unmet needs regarding “finding meaning out of your experience with your deceased family member’s illness” (*n* = 4/5, 80%) and “talking to others who have lost a loved one to the same illness (*n* = 3/5, 60%).

**Table 3:** Rating of unmet needs by adults and family caregivers of individuals with telomere biology disorders (*N* = 40).

	Adults (*n* = 19)			Caregivers (*n* = 16)			Bereaved ^a^ (*n* = 5)		
		Rating			Rating			Rating	
Unmet need	*n*	*M* (*SD*)	Range	*n*	*M* (*SD*)	Range	*n*	*M* (*SD*)	Range
**Psychosocial domain**	**19**	**5.9 (3.4)**	**0–11.6**	**15**	**5.4 (2.4)**	**1.0–9.5**	**4**	**5.2 (2.2)**	**3.0–8.0**
Dealing with your emotional distress	16	6.6 (5.0)	0–16.0	11	6.5 (3.0)	3.0–12.0	1	6.0 (0.0)	6.0–6.0
Helping them with their emotional distress	NA	NA	NA	12	7.3 (4.5)	0–16.0	NA	NA	NA
Dealing with emotional distress of family	NA	NA	NA	NA	NA	NA	2	6.0 (0.0)	6.0–6.0
Talking to anyone about your concerns	9	5.7 (3.1)	0–9.0	7	3.9 (1.2)	2.0–6.0	NA	NA	NA
Talking to them about their concerns	NA	NA	NA	10	3.9 (3.8)	0–12.0	NA	NA	NA
Talking about deceased family member’s illness	NA	NA	NA	NA	NA	NA	2	7.0 (1.4)	6.0–8.0
Talking to others who lost loved one to same illness	NA	NA	NA	NA	NA	NA	3	5.0 (3.6)	2.0–9.0
Finding meaning in your/their illness	7	7.0 (5.9)	0–16.0	3	8.3 (4.0)	4.0–12.0	4	4.0 (3.7)	0.0–9.0
Helping them/family find meaning in their illness	NA	NA	NA	4	3.8 (3.3)	0–8.0	2	5.0 (1.4)	4.0–6.0
Satisfied in caregiver-care receiver relationship	5	3.6 (2.6)	0–6.0	7	3.9 (3.1)	0–8.0	NA	NA	NA
Having an intimate relationship	5	8.0 (7.5)	0–16.0	6	6.7 (5.4)	0–16.0	0	Not selected	Not selected
Dealing with lifestyle changes	6	4.3 (3.0)	0–9.0	4	5.8 (2.4)	4.0–9.0	1	8.0 (0.0)	8.0–8.0
**Medical domain**	**18**	**6.5 (4.5)**	**0–15.2**	**15**	**5.1 (3.1)**	**0–10.0**	**NA**	**NA**	**NA**
Getting the best possible care for yourself/them	14	6.3 (4.8)	0–16.0	12	5.4 (3.8)	0–12.0	NA	NA	NA
Communicating with medical staff	11	5.8 (5.2)	0–16.0	11	3.3 (3.9)	0–12.0	NA	NA	NA
Getting involved in medical decision-making	9	5.3 (5.0)	0–12.0	8	4.3 (3.6)	0–8.0	NA	NA	NA
Getting information about illness	9	8.3 (5.4)	0–16.0	8	5.8 (4.3)	0–12.0	NA	NA	NA
Understanding/navigating insurance coverage	9	7.2 (7.0)	0–16.0	2	6.0 (2.8)	4.0–8.0	0	Not selected	Not selected
Managing your/their illness-related pain	6	5.8 (5.7)	0–16.0	5	7.0 (5.2)	3.0–16.0	NA	NA	NA
Managing your/their other symptoms (non-pain)	11	6.3 (4.0)	0–12.0	9	7.1 (5.3)	0–16.0	NA	NA	NA
**Financial domain**	**11**	**6.3 (5.1)**	**0–16.0**	**7**	**9.2 (6.2)**	**0–16.0**	**1**	**8.0 (0.0)**	**8.0–8.0**
Taking care of bills	9	6.3 (4.9)	0–16.0	5	11.6 (3.6)	6.0–16.0	1	8.0 (0.0)	8.0–8.0
Having enough insurance coverage	4	9.5 (7.5)	2.0–16.0	3	8.0 (8.0)	0–16.0	NA	NA	NA
Paying for your/their medical expenses	7	7.0 (6.9)	0–16.0	4	10.3 (6.4)	1.0–16.0	NA	NA	NA
**Daily activity domain**	**14**	**6.1 (2.8)**	**0–10.5**	**14**	**4.9 (2.0)**	**2.3–8.5**	**4**	**5.5 (2.3)**	**3.5–8.5**
Meeting your personal needs	7	5.4 (3.3)	0–9.0	8	5.6 (3.2)	2.0–12.0	1	6.0 (0.0)	6.0–6.0
Getting help from others to take time for self	NA	NA	NA	3	5.3 (1.2)	4.0–6.0	0	Not selected	Not selected
Taking time off work	4	6.8 (5.1)	0–12.0	7	6.6 (3.0)	2.0–9.0	0	Not selected	Not selected
Getting together with family and friends	4	4.8 (3.8)	0–9.0	6	5.5 (2.6)	3.0–9.0	2	5.5 (3.5)	3.0–8.0
Being satisfied with family/friend relationships	8	6.0 (3.7)	0–12.0	5	4.4 (2.7)	2.0–9.0	2	6.5 (3.5)	4.0–9.0
Balancing work or school with managing illness	8	7.8 (4.0)	0–12.0	6	4.8 (4.0)	0–12.0	NA	NA	NA
Reorganizing roles in family	2	5.0 (1.4)	4.0–6.0	3	3.3 (3.1)	0–6.0	1	4.0 (0.0)	4.0–4.0
Finding assistance with your/their daily needs	0	Not selected	Not selected	6	4.0 (6.0)	0–16.0	NA	NA	NA

NA indicates not ascertained. Mean scores >0 signify unfulfillment of a need, with higher (vs. lower) mean scores indicating relatively greater unfulfillment of a need. The lower and upper bounds of possible scores were 0 and 16, respectively. The emboldened domain mean scores were calculated by averaging the respective item scores for values > 0 under each domain (psychosocial, medical, financial, and daily activity).

^a^The survey version for bereaved individuals also included items not included on the versions for adults with TBDs and caregivers, respectively. No bereaved respondent selected these items as a need they had in the past four weeks: “understanding (navigating) the health care system,” “getting legal paperwork done,” taking part in your usual social/recreational activities,” and “getting help with your household activities.” One bereaved individual indicated that “taking care of your own health” was an extremely important need but one that they were extremely satisfied fulfilling (i.e., this need item was scored as 0 and, therefore, did not meet the definition of an unmet need).

In interviews, participants reported similar unmet psychosocial needs and elaborated on strategies they used to cope with emotional distress. TBD diagnoses, manifestations, prognoses, and chronic medical uncertainty often resulted in a range of dynamic emotions, including fear, sadness, anger, grief, and guilt (e.g., due to children inheriting TBDs). Many participants reported emotional problems, such as anxiety, depression, and post-traumatic stress disorder, that negatively impacted daily life.

It almost feels like I can’t remember a lot of [my bone marrow transplant] just because there is so much trauma in the last year and a half…When I was first diagnosed, there was so much unknown and just so much anxiety about all the different possibilities of things. (adult with a TBD, age 34)

Specific sources of emotional distress included a “lifetime of healthcare burden” and medical decision-making in the context of limited medical knowledge. Other stressors included transitioning children to adult care and coping with or discussing personal or family members’ illness-related symptoms, screenings, procedures, anticipation of loss, or bereavement.

Participants described a range of coping strategies, including psychological counseling, mind-body practices (e.g., breathwork), spending time with friends, and participating in faith or TBD communities/support groups. Several participants reported being intentional about enjoying life and setting boundaries around TBD information-seeking and distressing TBD-related conversations. However, these coping strategies sometimes presented their own challenges. For example, some participants described difficulties enjoying life while feeling like a “ticking time bomb,” feeling hope while bracing for the worst, and dealing with conflict in family relationships after attempting to set boundaries.

#### Medical needs

Nearly all adults with TBDs (*n* = 18/19, 95%) and current caregivers (*n* = 15/16, 94%) indicated an unmet medical need via the survey. The majority reported difficulty “getting the best possible care for yourself/them” (adults: *n* = 14/19, 74%; caregivers: *n* = 12/16, 75%), “communicating with medical staff” (adults: *n* = 11/19, 58%; caregivers: *n* = 11/16, 69%), and “managing your/their other symptoms (non-pain),” such as fatigue (adults: *n* = 11/19, 58%; caregivers: *n* = 9/16, 56%). “Getting information about illness” and “understanding/navigating insurance coverage” were among the highest rated unmet needs reported by both groups (Table 3). Both groups also reported difficulties “getting involved in medical decision-making” and “managing your/their illness-related pain.”

Interview participants detailed numerous interrelated challenges and perceived barriers to meeting medical needs. Challenges receiving optimal care for complex TBD manifestations included lack of TBD knowledge and expertise within the medical community, difficulties with accessing specialists, transitioning from pediatric to adult care, lack of coordinated care, and multi-year delays in diagnosis. Disruptions in receiving optimal care occurred due to time spent navigating complicated healthcare system policies and insurance coverage for major procedures (e.g., organ transplant) and recommended routine screenings (e.g., cancer screenings).

It has been my recent experience in seeking out a lung transplant that there is a reluctance for many healthcare centers to take on complicated cases…I have been rejected at 4 big centers…For me this has been a huge stress… (adult with a TBD, age 52)

Interactions with medical staff or insurance companies that left participants feeling unheard, dismissed, stigmatized, unsafe, or uninformed often triggered development of strong beliefs regarding the need to advocate for self or family members and to seek (or provide others in the TBD community with) TBD-related information and social support. For many, partnering with healthcare providers who would better meet their/their family members’ needs required traveling long distances or moving. Most participants also reported attempting to fulfill medical information and support needs through TBD- or disease-specific patient/family advocacy organizations and through educational resources, namely the *Telomere Biology Disorders: Diagnosis and Management Guidelines* [13].

Additional medical challenges described by participants included self-managing and/or helping family members manage numerous and dynamic multi-organ system effects of TBDs (e.g., fatigue, shortness of breath, pain) and acute or chronic effects of procedures (e.g., post-transplant graft-versus-host disease) and medications (e.g., osteoporotic fractures). Participants reported feeling burdened by the immense responsibility of being the “gatekeeper” of their own and/or family members’ health within healthcare settings (e.g., making life-altering medical decisions), at home, and in the community (e.g., protecting from infection/communicable diseases). The COVID-19 pandemic magnified this burden and raised concerns about actual or feared delays in routine, and potentially lifesaving, appointments, screenings, and procedures.

#### Financial needs

The majority of adults with TBDs (*n* = 11/19, 58%), several family caregivers (*n* = 7/16, 44%), and a bereaved caregiver (*n* = 1/5, 20%) reported unmet financial needs via the survey. The mean scores for all individual items pertaining to unmet financial needs were among the highest in the needs assessment survey data. These unmet needs included “taking care of bills,” “having enough insurance coverage,” and “paying for your/their medical expenses” (Table 3).

In interviews, several participants described a range of financial difficulties due to TBDs, despite being medically insured. Some participants with insurance reported that copays for frequent medical appointments (e.g., 3–4 per month), treatments, and/or major procedures caused financial insecurity. Financial strain due to medical care costs had daily and long-term effects on other financial needs for several participants, including challenges affording food, non-medical bills, and one’s own or children’s college or vocational training. Some reported insufficient insurance coverage was among the most distressing aspects of living with a TBD.

We are constantly preparing in a different way…My husband, he’s working more. I’m working more… [Our son with a TBD] does his bone marrow [biopsy] every year…And we have a copay…it’s still a lot. 20 percent of a lot is still a lot. We’re kind of building up finances to pay for the things that are going to be required of that.” (caregiver, age 27)

#### Daily activity needs

Most adults with TBDs (*n* = 14/19, 74%), current caregivers (*n* = 14/16, 88%), and bereaved caregivers (*n* = 4/5, 80%) indicated unmet daily activity needs (Table 3). Similar to the financial domain, unmet daily activity needs were reported relatively less frequently than in psychosocial and medical domains. However, mean scores were relatively high for “balancing work or school with managing illness” (adults with TBDs) and “being satisfied with family/friend relationships” (adults with TBDs and bereaved caregivers). In addition, for both adults with TBDs and current caregivers, mean scores indicated “taking time off work” is an important need, but one that is unsatisfactorily fulfilled. Other unmet needs for all groups included “meeting personal needs,” “getting together with family and friends,” and “reorganizing family roles.” Caregivers also reported “getting help from others to take time for oneself” and “finding assistance with daily needs” for their family member(s) with a TBD were unfulfilled needs.

Interview participants described several challenges fulfilling daily activity needs that corroborated survey data. Permanent leaves of absence from work were often required to convalesce, provide care to sick or recovering family, or to grieve a TBD-related family death. Some participants changed jobs or careers to protect self or family from acquiring infection/communicable diseases or to allow more time for self-care, life enjoyment, or family time. Participants described shifts in work life as profoundly distressing, especially in unsupportive work environments or when job/career loss led to changes in medical insurance coverage or had negative effects on self-identity.

Family caregivers reported their children with TBDs experienced frequent and, often, long-term absences from school, many missing entire grades to recover from acute illness, procedures, and chronic complications. Caregivers described the practical, emotional, and social burdens of insufficient school accommodations, homeschooling due to illness or infection risk, and frequent isolation from school friends and communities. They also reported concerns that TBD-related illness and symptoms, such as fatigue and shortness of breath, impacted their children’s intellectual, physical, and social development (e.g., attention, running, play, development of friendships). Family caregivers of children (or spouses) with a TBD also mentioned difficulty finding time to meet their own personal needs, including health maintenance behaviors, such as exercise, routine medical care, and mental health counseling.

I’m always still balancing preparing for something and actually living my life…One of the things that I stopped doing when [my son with a TBD] got sick is…I stopped running because he couldn’t run…And so, I just couldn’t enjoy running anymore. I couldn’t enjoy my own sort of physical health. (caregiver, age 54)

Interview participants reported unmet needs regarding forming and maintaining supportive relationships with family, friends, and romantic partners. Several reported TBD-related illness or the need to protect oneself and/or family members from infection limited their ability to gather with family, friends, and other social networks (e.g., faith communities), which resulted in isolation and feelings of loneliness. TBD- and disease-specific patient/family advocacy organizations played an important role in emotional support and fostering feelings of connection with others in similar circumstances.

## Discussion

Medical complexity and uncertainty surrounding TBDs and most other rare diseases often result in a spectrum of unmet medical and psychosocial needs. This mixed methods study quantitatively and qualitatively assessed and identified numerous unmet needs in multiple life domains faced by adults and family caregivers of individuals with TBDs. Several similar issues (e.g., distress, care coordination) and gaps in addressing medical and psychosocial needs have been reported in the literature on individuals [6–8] and families [9, 17–20] affected by rare genetic diseases. Gaps in meeting these needs may pose significant psychosocial risks (e.g., anxiety, depression) that affect healthcare outcomes, quality of life, and psychological well-being.

Need item scores were similar within and across survey domains for adults with TBDs and current caregivers. Bereaved caregivers also identified several TBD-related unmet needs. These findings suggest the importance of assessing and addressing illness-related needs at the level of the family system. Interview data also illuminated the importance of evaluating and supporting an especially vulnerable group: family caregivers of individuals with TBDs who also have a personal TBD diagnosis. Notably, family caregivers had relatively higher average need scores across all four domains studied (psychosocial, medical, financial, and daily activities) when compared with past studies of family caregivers of individuals with cancer [24].

Our data are also congruent with findings previously reported among individuals and families with FA [17–20], including chronic uncertainty and emotional distress associated with managing and coordinating care for a rare disease with multisystemic complications. Our study findings, along with past research on individuals with rare disease and caregiver populations [20, 24, 29], suggest that individuals with rare disease and family caregivers may benefit from routine psychosocial screening and referrals for assistance (e.g., mental health counselors, support groups), including bereavement.

Strengths of our study include use of mixed method and participatory design approaches involving a TBD community. Triangulation of qualitative interview data with quantitative survey data provided important context regarding unmet needs, enhancing the conclusions and clinical and research implications. Despite these strengths, there were also limitations. Quantitative analysis was limited by self-reported, cross-sectional survey data from a small sample of participants. Given the manner in which mean scores were calculated on the needs assessment, we were unable to identify needs that might have been well-addressed or met. In addition, participants were predominantly female, English-speaking, highly educated, and medically insured. Consequently, our findings miss the perspectives of individuals with non-female gender identities as well as those with less English proficiency, lower education, and/or no health insurance who may face even more profound and complex unmet needs, such as poor access to local resources that facilitate initiation of care [5]. Recruitment through an existing clinical trial and patient/family organization also limits broad generalizability of our findings to the entire TBD population.

Our findings are likely highly relevant to individuals and families with other complex rare diseases. Most individuals with rare disease have a long diagnostic journey, multiple medical problems, and limited (or no) access to expert medical care [4–7, 15]. Further, this study provides clinicians with a foundation for understanding complex and dynamic TBD-related needs; we recommend considering these factors across similarly complex (even if different) rare diseases. We recommend psychosocial risk assessment and management in addition to medical risk management for adults and family caregivers of individuals with TBDs. The multisystemic nature of TBDs makes transdisciplinary communication critically important for coordination of care, medical decision-making, and satisfaction and trust in the patient-provider (-caregiver) relationship.

## Supplementary information

Supplementary Material 1

Supplementary Material 2

Supplementary Material 3

## Data Availability

De-identified data will be shared with qualified investigators after IRB review and establishment of appropriate data transfer agreements. Please contact the corresponding author for data requests.
