# Supplementary material for: Defining the complex needs of families with rare diseases—the example of telomere biology disorders
Source: Eur J Hum Genet. 2024 Oct 1;32(12):1615–23. doi: 10.1038/s41431-024-01697-6 (PMC11607413; doi:10.1038/s41431-024-01697-6)
Supplement: Supplementary file 1 — Supplementary Material 1 [file 41431_2024_1697_MOESM1_ESM.pdf]

Supplementary Material 1: Clinical features of telomere biology disorders and health care recommendations. Table adapted from Savage.[1] Summary of clinical features adapted from Niewisch and Savage[2] and Niewisch et al.[3] Surveillance and management recommendations are based on expert opinion as published.[4,5]

Organ system	Features	Surveillance	Management
**Mucocutaneous**	Classic triad: Nail dysplasia, abnormal skin pigmentation (hyper/hypopigmentation), oral leukoplakia<br>Additional features: Premature greying, scalp or eyelash hair loss, adermatoglyphia, palmoplantar hyperkeratosis, hyperhidrosis, epiphora, blepharitis	Regular skin self-examination and annual full body skin exam by dermatologist	Regular use of sunscreen, advise to avoid excessive sun exposure
**Hematologic**	Cytopenias, bone marrow failure[#], isolated aplastic anemia	Baseline CBC, bone marrow aspiration and biopsy with careful morphologic examination and cytogenetic studies (G-banding and FISH).<br>If CBCs are normal and stable, annual CBC to identify trends and early manifestations. Annual bone marrow evaluation based on clinical features. Consider next generation somatic sequencing of bone marrow.	Early referral to hematopoietic cell transplant center with expertise in TBDs<br>Participation in a clinical trial is encouraged<br>Consider oral androgens, such as danazol, for cytopenias. Improvement in cytopenias may take 4-8 weeks of androgens<br><u>If on androgen therapy:</u> CBCs prior to therapy, repeat CBCs every 4-6 weeks to assess response, when counts are stable every 2-3 months<br>Monitor more frequently if cytopenias change

			or a cytogenetic clone is identified.
**Immunologic**	Immunodeficiency (lymphopenia, decreased, T, B and NK cell count, hypogammaglobulinemia)	Consider a complete immunological evaluation including lymphocyte subsets, lymphocyte proliferation response, serum IgG, IgM, IgA levels, childhood vaccine antibody titers	Follow-up according to clinical features<br>Childhood vaccines, including human papilloma virus and influenza, if not contraindicated due to immunodeficiency or HCT
**Central Nervous System**	Brain structure: Microcephaly, cerebellar hypoplasia/atrophy, intracranial calcifications, intracranial cysts<br>Neurological: Learning difficulty, developmental delay (psychomotor and mental), ataxia<br>Psychiatric: mood disorders, schizophrenia	MRI assessment for cerebellar hypoplasia at diagnosis in children or individuals with developmental delay or learning problems.	Regular evaluation for developmental delay and early intervention if needed<br>Assess for mood disorders and related illnesses
**Ophthalmologic**	Lacrimal duct stenosis, epiphora, blepharitis, entropion, trichiasis, keratoconjunctivitis, cataracts, ulcers<br>Retinal abnormalities: retinal detachment, pigmentary changes, exudative retinopathy, proliferative retinopathy<br>Corneal limbal insufficiency	Annual examination to detect/correct vision problems, abnormally growing eyelashes, lacrimal duct stenosis, retinal changes, bleeding, cataracts, and glaucoma	
**Otolaryngology**	Oral leukoplakia and high risk of HNSCC<br>Reduced hearing reported	Annual cancer screening by a dentist and an otolaryngologist beginning in adolescence. Patient should be taught how to perform a monthly self-examination for	Follow oral leukoplakia carefully and biopsy any changes or suspicious sites.

		oral, head and neck cancer<br>Baseline hearing evaluation	
**Dental**	Oral leukoplakia and high risk of HNSCC<br>Caries, periodontitis, decreased crown/root ratio, taurodontism[6,7]	Dental hygiene and screening every 6 months Maintain good oral hygiene<br>Inform the primary dentist of the patient's increased risk of HNSCC	
**Cardiac**	Atrial septal defect, ventricular septal defect, dilated cardiomyopathy, reported but not common	Baseline evaluation for arteriovenous malformations and cardiac malformations. Bubble echocardiogram for pulmonary symptoms in the absence of pulmonary fibrosis	
**Pulmonary**	Pulmonary fibrosis, hepatopulmonary syndrome, pulmonary arteriovenous malformations, interstitial pneumonitis, hypersensitivity pneumonitis, pleuroparenchymal fibroelastosis, pulmonary emphysema, combined pulmonary fibrosis and emphysema	Baseline PFTs at diagnosis and annually, beginning at an age when the patient can properly perform the test<br>Counsel patients to avoid exposure to cigarette smoke	Early referral to specialist for shortness of breath or unexplained cough<br>Antifibrotics for pulmonary fibrosis require further study in TBDs. Clinical trial participation is encouraged
**Gastrointestinal and liver**	Esophageal narrowing/stricture/webs, dysphagia, failure to thrive, enteropathy/enterocolitis[8]<br>Noninfectious/nonalcoholic liver fibrosis/cirrhosis, nodular regenerative hyperplasia/non-cirrhotic portal hypertension	Evaluate for clinical history suspicious for esophageal stenosis and/or enteropathy and refer as needed<br>Liver function tests at least annually<br>Upper and/or lower endoscopy based on symptoms	<u>If on androgen therapy:</u><br>Check liver function tests prior to starting, then every 6-12 weeks<br>Check lipid profile prior to starting and every 6-12 months

			Perform liver ultrasound examination prior to initiation of androgens and semiannually to evaluate for adenomas, carcinomas or fibrosis
**Vascular**	Gastrointestinal telangiectatic anomalies and GI bleeding, pulmonary arteriovenous malformations, retinal vessel abnormalities	Assess for GI bleeding and PAVM as above<br>Ophthalmic evaluations as above	Early referral to experts in vascular anomalies. Propranolol and bevacizumab have been tried in a few patients but not systematically studied[9]
**Genitourinary**	Urethral stenosis/strictures/phimosis, undescended testes (rare), males: hypospadias, penile leukoplakia, females: urethral stricture, vaginal atrophy, and leukoplakia	Baseline assessment for genitourinary anomalies, including symptoms of urethral stenosis	Assess for signs or symptoms of urethral stenosis
**Reproductive**	Infertility reported in one case study, normal fertility in women with DC/TBD analyzed for pregnancy related complications	Annual gynecologic evaluation with HPV testing starting at 18 years of age or at start of sexual activity<br>Referral to maternal fetal medicine specialist for high-risk pregnancy	
**Endocrine**	Short stature, hypogonadism (male) reported by not common	Follow growth carefully	<u>If on androgen therapy:</u> Evaluation prior to therapy, on treatment regular (annually) evaluation for side effects

**Skeletal**	Osteopenia, osteoporosis, avascular necrosis of hips and shoulders[10]	Baseline bone density scan to evaluate for osteopenia at approximately 14 years of age. Follow-up bone density scans yearly or as recommended by physician. Evaluation of hip and shoulder avascular necrosis based on symptoms	Vitamin D and calcium as needed to optimize bone health<br><br><u>If on androgen therapy:</u> In growing child baseline prior to treatment, then every 6-12 months
**Other**	Intrauterine growth retardation, low birthweight[7]	Evaluations for developmental delay as above	
**Reported malignancies**	AML, MDS, HNSCC (especially tongue), NHL, anal SCC, Skin: BCC, SCC<br><br>Reported but rare: esophagus, rectal adenocarcinomas, cervix, thyroid, Hodgkin lymphoma, PTLD[11], lung, stomach, pancreas, colon, hepatic adenoma, hepatic angiosarcoma	Annual evaluations by organ system as above	Increased sensitivity to therapeutic radiation and chemotherapy may require dose reductions

Abbreviations: AML, acute myeloid leukemia; BCC, basal cell carcinoma; CBC, complete blood count; HCT, hematopoietic cell transplantation; HNSCC, head and neck squamous cell carcinoma; MDS, myelodysplastic syndrome; NHL, non-Hodgkin lymphoma; PAVM, pulmonary arteriovenous malformation; PFTs, pulmonary function tests; PTLD, post-transplant lymphoproliferative disease; SCC, squamous cell carcinoma

[#] <u>Classification of bone marrow failure</u>
Mild: ANC 1000-<1500/mm3, Platelets 50.000-<150.000/mm3, Hb ≥8g/dl-less than normal for age
Moderate: ANC 500-<1000/mm3, Platelets 20.000-< 50.0000/mm3, Hb ≥8g/dl-less than normal for age
Severe: ANC < 500/mm3, Platelets < 20.000/mm3, Hb < 8.0 g/dl

References:

1. Savage SA. Dyskeratosis congenita and telomere biology disorders. *Hematology Am Soc Hematol Educ Program*. Dec 9 2022;2022(1):637-648. doi:10.1182/hematology.2022000394
2. Niewisch MR, Savage SA. An update on the biology and management of dyskeratosis congenita and related telomere biology disorders. *Expert Rev Hematol*. Dec 2019;12(12):1037-1052. doi:10.1080/17474086.2019.1662720

3. Niewisch MR, Giri N, McReynolds LJ, Alsaggaf R, Bhala S, Alter BP, Savage SA. Disease progression and clinical outcomes in telomere biology disorders. *Blood*. Mar 24 2022;139(12):1807-1819. doi:10.1182/blood.2021013523
4. *Telomere Biology Disorders Diagnosis and Management Guidelines*. Team Telomere, Inc. ; 2022:509.
5. Walsh MF, Chang VY, Kohlmann WK, et al. Recommendations for Childhood Cancer Screening and Surveillance in DNA Repair Disorders. *Clinical Cancer Research*. 2017;23(11):e23-e31. doi:10.1158/1078-0432.Ccr-17-0465
6. Atkinson JC, Harvey KE, Domingo DL, et al. Oral and dental phenotype of dyskeratosis congenita. *Oral Dis*. Jul 2008;14(5):419-27.
7. Dokal I. Dyskeratosis congenita in all its forms. *British Journal of Haematology*. 2000;110(4):768-779. doi:10.1046/j.1365-2141.2000.02109.x
8. Jonassaint NL, Guo N, Califano JA, Montgomery EA, Armanios M. The gastrointestinal manifestations of telomere-mediated disease. *Aging Cell*. Apr 2013;12(2):319-23. doi:10.1111/acel.12041
9. Higgs C, Crow YJ, Adams DM, et al. Understanding the evolving phenotype of vascular complications in telomere biology disorders. *Angiogenesis*. Feb 2019;22(1):95-102. doi:10.1007/s10456-018-9640-7
10. Savage SA. Chapter Two - Human Telomeres and Telomere Biology Disorders. In: Calado RT, ed. *Progress in Molecular Biology and Translational Science*. Academic Press; 2014:41-66.
11. Alter BP, Giri N, Savage SA, Rosenberg PS. Cancer in the National Cancer Institute inherited bone marrow failure syndrome cohort after fifteen years of follow-up. *Haematologica*. Jan 2018;103(1):30-39. doi:10.3324/haematol.2017.178111
