# Supplementary material for: Defining the complex needs of families with rare diseases—the example of telomere biology disorders
Source: Eur J Hum Genet. 2024 Oct 1;32(12):1615–23. doi: 10.1038/s41431-024-01697-6 (PMC11607413; doi:10.1038/s41431-024-01697-6)
Supplement: Supplementary file 2 — Supplementary Material 2 [file 41431_2024_1697_MOESM2_ESM.pdf]

Supplementary Material 2: Needs assessment survey items

Next, we will ask you several questions about your needs related to DC or a related TBD. Based on your responses to previous questions, more than one option may display below. If more than one option is displayed, please choose one option and complete the rest of this survey from that one perspective.

I will complete the survey from the perspective of:

- ○ a person with DC or a related TBD
- ○ a caregiver of someone living with DC or a related TBD
- ○ a bereaved parent or legal guardian of a child who had DC or a related TBD
- ○ a bereaved spouse or partner of someone who had DC or a related TBD

Display This Question:

If Next, we will ask you several questions about your needs related to DC or a related TBD. Based on... = a person with DC or a related TBD

We are interested in your current needs and concerns related to DC or a related TBD so we understand how health care providers may better assist you.

- The following questions ask about whether or not you have experienced certain needs during the past 4 weeks.
- For each need you indicate having, you will be asked how important the need has been for you during the past 4 weeks.
- You also will be asked how satisfied you have been with the fulfillment of the need during the past 4 weeks.
- Finally, you will be asked how much your needs have changed during the COVID-19 pandemic.

Display This Question:

If Next, we will ask you several questions about your needs related to DC or a related TBD. Based on... = a person with DC or a related TBD

During the past 4 weeks, have you experienced a need related to any of the following?

Mark all that apply.

- ☐ Dealing with emotional distress (e.g., anger, anxiety, depression, fear, resentment, etc.)
- ☐ Getting the best possible care for yourself
- ☐ Taking care of bills
- ☐ Meeting your personal needs
- ☐ Communicating with medical staff
- ☐ Having enough insurance coverage
- ☐ Talking to anyone about your concerns
- ☐ Getting involved in medical decisions affecting you
- ☐ Paying for your medical expenses
- ☐ Taking time off from work
- ☐ Finding meaning out of your experience with your illness
- ☐ Getting information about the illness you were diagnosed with (e.g., prognosis, treatment, side effects, nutrition)
- ☐ Getting together with family and friends
- ☐ Understanding or navigating medical or insurance coverage
- ☐ Being satisfied with your relationship with other family members and friends

- [ ] Managing your illness-related pain
- [ ] Managing your other illness-related symptoms (e.g., fatigue, nausea)
- [ ] Balancing work or school
- [ ] Being satisfied with relationships with people who provide your care
- [ ] Reorganizing roles among family members
- [ ] Having an intimate relationship
- [ ] Dealing with lifestyle changes
- [ ] Finding assistance with daily needs (e.g., preparing meals, transportation, etc.)

Display This Question:

If Next, we will ask you several questions about your needs related to DC or a related TBD. Based on... = a person with DC or a related TBD

And And During the past 4 weeks, have you experienced a need related to any of the following? Mark all... q://QID23/SelectedChoicesCount Is Greater Than or Equal to 1

Carry Forward Selected Choices from "During the past 4 weeks, have you experienced a need related to any of the following? Mark all that apply."

During the past 4 weeks, **how important** was the need related to…

	Not at all	A little bit	Somewhat	Quite a bit	Extremely
Dealing with emotional distress (e.g., anger, anxiety, depression, fear, resentment, etc.)	◯	◯	◯	◯	◯
Getting the best possible care for yourself	◯	◯	◯	◯	◯
Taking care of bills	◯	◯	◯	◯	◯
Meeting your personal needs	◯	◯	◯	◯	◯
Communicating with medical staff	◯	◯	◯	◯	◯
Having enough insurance coverage	◯	◯	◯	◯	◯
Talking to anyone about your concerns	◯	◯	◯	◯	◯
Getting involved in medical decisions affecting you	◯	◯	◯	◯	◯
Paying for your medical expenses	◯	◯	◯	◯	◯
Taking time off from work	◯	◯	◯	◯	◯
Finding meaning out of your experience with your illness	◯	◯	◯	◯	◯

Getting information about the illness you were diagnosed with (e.g., prognosis, treatment, side effects, nutrition)	○	○	○	○	○
Getting together with family and friends	○	○	○	○	○
Understanding or navigating medical or insurance coverage	○	○	○	○	○
Being satisfied with your relationship with other family members and friends	○	○	○	○	○
Managing your illness-related pain	○	○	○	○	○
Managing your other illness-related symptoms (e.g., fatigue, nausea)	○	○	○	○	○
Balancing work or school	○	○	○	○	○
Being satisfied with relationships with people who provide your care	○	○	○	○	○
Reorganizing roles among family members	○	○	○	○	○

Having an intimate relationship	○	○	○	○	○
Dealing with lifestyle changes	○	○	○	○	○
Finding assistance with daily needs (e.g., preparing meals, transportation, etc.)	○	○	○	○	○

Display This Question:

If Next, we will ask you several questions about your needs related to DC or a related TBD. Based on... = a person with DC or a related TBD

And And During the past 4 weeks, have you experienced a need related to any of the following?   Mark all... q://QID23/SelectedChoicesCount Is Greater Than or Equal to 1

Carry Forward Selected Choices from "During the past 4 weeks, have you experienced a need related to any of the following?   Mark all that apply."

During the past 4 weeks, how satisfied have you been with fulfilling the need to deal with...

	Not at all	A little bit	Somewhat	Quite a bit	Extremely
Dealing with emotional distress (e.g., anger, anxiety, depression, fear, resentment, etc.)	○	○	○	○	○
Getting the best possible care for yourself	○	○	○	○	○
Taking care of bills	○	○	○	○	○
Meeting your personal needs	○	○	○	○	○
Communicating with medical staff	○	○	○	○	○
Having enough insurance coverage	○	○	○	○	○
Talking to anyone about your concerns	○	○	○	○	○
Getting involved in medical decisions affecting you	○	○	○	○	○
Paying for your medical expenses	○	○	○	○	○
Taking time off from work	○	○	○	○	○
Finding meaning out of your experience with your illness	○	○	○	○	○

Getting information about the illness you were diagnosed with (e.g., prognosis, treatment, side effects, nutrition)	○	○	○	○	○
Getting together with family and friends	○	○	○	○	○
Understanding or navigating medical or insurance coverage	○	○	○	○	○
Being satisfied with your relationship with other family members and friends	○	○	○	○	○
Managing your illness-related pain	○	○	○	○	○
Managing your other illness-related symptoms (e.g., fatigue, nausea)	○	○	○	○	○
Balancing work or school	○	○	○	○	○
Being satisfied with relationships with people who provide your care	○	○	○	○	○
Reorganizing roles among family members	○	○	○	○	○

Having an intimate relationship	○	○	○	○	○
Dealing with lifestyle changes	○	○	○	○	○
Finding assistance with daily needs (e.g., preparing meals, transportation, etc.)	○	○	○	○	○

Display This Question:

If Next, we will ask you several questions about your needs related to DC or a related TBD. Based on... = a caregiver of someone living with DC or a related TBD

We are interested in your current needs and concerns related to DC or a related TBD so we understand how health care providers may better assist you. The following list contains concerns expressed by other individuals who have provided care for ill family members or friends.

- The following questions ask about whether or not you have experienced certain needs during the past 4 weeks.
- For each need you indicate having, you will be asked how important the need has been for you during the past 4 weeks.
- You also will be asked how satisfied you have been with the fulfillment of the need during the past 4 weeks.
- Finally, you will be asked how much your needs have changed during the COVID-19 pandemic.

For items containing him/her/them, his/her/their, or he/she/they, in your mind please refer to the name or names of the person or people you provide care for.

Display This Question:

If Next, we will ask you several questions about your needs related to DC or a related TBD. Based on... = a caregiver of someone living with DC or a related TBD

During the past 4 weeks, have you experienced a need related to any of the following?

Mark all that apply.

- ☐ Helping his/her/their emotional distress (e.g., anger, anxiety, depression, fear, resentment, etc.)
- ☐ Getting the best possible care him/her/them
- ☐ Taking care of bills
- ☐ Meeting your personal needs
- ☐ Dealing with your emotional distress (e.g., anger, anxiety, depression, fear, resentment, etc.)
- ☐ Communicating with his/her/their medical staff
- ☐ Having enough insurance coverage for him/her/them
- ☐ Getting help from others in order to take time for yourself
- ☐ Talking to him/her/them about his/her/their concerns
- ☐ Talking to anyone about your concerns
- ☐ Getting involved in medical decisions affecting him/her/them
- ☐ Paying for his/her/their medical expenses
- ☐ Taking time off from work
- ☐ Finding meaning out of your experience with his/her/their illness
- ☐ Getting information about the illness he/she/they was/were diagnosed with (e.g., prognosis, treatment, side effects, nutrition)
- ☐ Getting together with family and friends

- [ ] Helping him/her/them find meaning out of their illness
- [ ] Understanding/navigating medical and/or insurance coverage
- [ ] Being satisfied with your relationship with other family members and friends
- [ ] Managing his/her/their illness-related pain
- [ ] Managing his/her/their other illness-related symptoms (e.g., fatigue, nausea)
- [ ] Balancing work or school with caring for him/her/them
- [ ] Being satisfied with your relationship with him/her/them
- [ ] Reorganizing roles among family members
- [ ] Having an intimate relationship
- [ ] Dealing with lifestyle changes
- [ ] Assisting with his/her/their daily needs (e.g., preparing meals, transportation, etc.)

Display This Question:

If Next, we will ask you several questions about your needs related to DC or a related TBD. Based on... = a caregiver of someone living with DC or a related TBD

And And During the past 4 weeks, have you experienced a need related to any of the following? Mark all... q://QID54/SelectedChoicesCount Is Greater Than or Equal to 1

Carry Forward Selected Choices from "During the past 4 weeks, have you experienced a need related to any of the following? Mark all that apply."

During the past 4 weeks, **how important** was the need related to...

	Not at all	A little bit	Somewhat	Quite a bit	Extremely
Helping his/her/their emotional distress (e.g., anger, anxiety, depression, fear, resentment, etc.)	○	○	○	○	○
Getting the best possible care him/her/them	○	○	○	○	○
Taking care of bills	○	○	○	○	○
Meeting your personal needs	○	○	○	○	○
Dealing with your emotional distress (e.g., anger, anxiety, depression, fear, resentment, etc.)	○	○	○	○	○
Communicating with his/her/their medical staff	○	○	○	○	○
Having enough insurance coverage for him/her/them	○	○	○	○	○
Getting help from others in order to take time for yourself	○	○	○	○	○
Talking to him/her/them about his/her/their concerns	○	○	○	○	○
Talking to anyone about your concerns	○	○	○	○	○
Getting involved in medical decisions affecting him/her/them	○	○	○	○	○
Paying for his/her/their medical expenses	○	○	○	○	○
Taking time off from work	○	○	○	○	○
Finding meaning out of your experience with his/her/their illness	○	○	○	○	○

Getting information about the illness he/she/they was/were diagnosed with (e.g., prognosis, treatment, side effects, nutrition)	○	○	○	○	○
Getting together with family and friends	○	○	○	○	○
Helping him/her/them find meaning out of their illness	○	○	○	○	○
Understanding/navigating medical and/or insurance coverage	○	○	○	○	○
Being satisfied with your relationship with other family members and friends	○	○	○	○	○
Managing his/her/their illness-related pain	○	○	○	○	○
Managing his/her/their other illness-related symptoms (e.g., fatigue, nausea)	○	○	○	○	○
Balancing work or school with caring for him/her/them	○	○	○	○	○
Being satisfied with your relationship with him/her/them	○	○	○	○	○
Reorganizing roles among family members	○	○	○	○	○
Having an intimate relationship	○	○	○	○	○
Dealing with lifestyle changes	○	○	○	○	○
Assisting with his/her/their daily needs (e.g., preparing meals, transportation, etc.)	○	○	○	○	○

Display This Question:

If Next, we will ask you several questions about your needs related to DC or a related TBD. Based on... = a caregiver of someone living with DC or a related TBD

And And During the past 4 weeks, have you experienced a need related to any of the following? Mark all... q://QID54/SelectedChoicesCount Is Greater Than or Equal to 1

Carry Forward Selected Choices from "During the past 4 weeks, have you experienced a need related to any of the following? Mark all that apply."

During the past 4 weeks, how satisfied have you been with fulfilling the need to deal with...

	Not at all	A little bit	Somewhat	Quite a bit	Extremely
Helping his/her/their emotional distress (e.g., anger, anxiety, depression, fear, resentment, etc.)	○	○	○	○	○
Getting the best possible care him/her/them	○	○	○	○	○
Taking care of bills	○	○	○	○	○
Meeting your personal needs	○	○	○	○	○
Dealing with your emotional distress (e.g., anger, anxiety, depression, fear, resentment, etc.)	○	○	○	○	○
Communicating with his/her/their medical staff	○	○	○	○	○
Having enough insurance coverage for him/her/them	○	○	○	○	○
Getting help from others in order to take time for yourself	○	○	○	○	○
Talking to him/her/them about his/her/their concerns	○	○	○	○	○
Talking to anyone about your concerns	○	○	○	○	○
Getting involved in medical decisions affecting him/her/them	○	○	○	○	○
Paying for his/her/their medical expenses	○	○	○	○	○
Taking time off from work	○	○	○	○	○
Finding meaning out of your experience with his/her/their illness	○	○	○	○	○

Getting information about the illness he/she/they was/were diagnosed with (e.g., prognosis, treatment, side effects, nutrition)	○	○	○	○	○
Getting together with family and friends	○	○	○	○	○
Helping him/her/them find meaning out of their illness	○	○	○	○	○
Understanding/navigating medical and/or insurance coverage	○	○	○	○	○
Being satisfied with your relationship with other family members and friends	○	○	○	○	○
Managing his/her/their illness-related pain	○	○	○	○	○
Managing his/her/their other illness-related symptoms (e.g., fatigue, nausea)	○	○	○	○	○
Balancing work or school with caring for him/her/them	○	○	○	○	○
Being satisfied with your relationship with him/her/them	○	○	○	○	○
Reorganizing roles among family members	○	○	○	○	○
Having an intimate relationship	○	○	○	○	○
Dealing with lifestyle changes	○	○	○	○	○
Assisting with his/her/their daily needs (e.g., preparing meals, transportation, etc.)	○	○	○	○	○

Display This Question:

If Next, we will ask you several questions about your needs related to DC or a related TBD. Based on... = a bereaved parent or legal guardian of a child who had DC or a related TBD

OR If Next, we will ask you several questions about your needs related to DC or a related TBD. Based on... = a bereaved spouse or partner of someone who had DC or a related TBD

We are interested in your current needs and concerns related to DC or a related TBD so we understand how health care providers may better assist you. The following list contains concerns expressed by other individuals who have lost a family member.

- The following questions ask about whether or not you have experienced certain needs during the past 4 weeks.
- For each need you indicate having, you will be asked how important the need has been for you during the past 4 weeks.
- You also will be asked how satisfied you have been with the fulfillment of the need during the past 4 weeks.
- Finally, you will be asked how much your needs have changed during the COVID-19 pandemic.

Display This Question:

If Next, we will ask you several questions about your needs related to DC or a related TBD. Based on... = a bereaved parent or legal guardian of a child who had DC or a related TBD

OR If Next, we will ask you several questions about your needs related to DC or a related TBD. Based on... = a bereaved spouse or partner of someone who had DC or a related TBD

During the past 4 weeks, have you experienced a need related to any of the following?

Mark all that apply.

- ☐ Taking part in your usual social or recreational activities
- ☐ Talking to others who have lost a loved one to the same illness
- ☐ Reorganizing roles among family members
- ☐ Taking care of bills
- ☐ Talking with other people about your deceased family member's illness
- ☐ Dealing with emotional distress of other family members (e.g., anger, anxiety, depression, fear, resentment, etc.)
- ☐ Finding meaning out of your experience with your deceased family member's illness
- ☐ Getting help with your household activities
- ☐ Understanding medical or insurance coverage
- ☐ Being satisfied with your relationship with other family members and friends
- ☐ Taking care of your own health
- ☐ Dealing with lifestyle changes
- ☐ Dealing with emotional distress (e.g., anger, anxiety, depression, fear, resentment, etc.)
- ☐ Getting help from others in order to take time for yourself
- ☐ Meeting your personal needs
- ☐ Getting together with family and friends
- ☐ Taking time off from work

- [ ] Understanding or navigating the health care system
- [ ] Having an intimate relationship
- [ ] Helping other family members find meaning out of your deceased family member's illness
- [ ] Getting legal paperwork done

Display This Question:

If Next, we will ask you several questions about your needs related to DC or a related TBD. Based on... = a bereaved parent or legal guardian of a child who had DC or a related TBD

And And During the past 4 weeks, have you experienced a need related to any of the following?   Mark all... q://QID57/SelectedChoicesCount Is Greater Than or Equal to 1

Carry Forward Selected Choices from "During the past 4 weeks, have you experienced a need related to any of the following?   Mark all that apply."

During the past 4 weeks, **how important** was the need related to...

	Not at all	A little bit	Somewhat	Quite a bit	Extremely
Taking part in your usual social or recreational activities	○	○	○	○	○
Talking to others who have lost a loved one to the same illness	○	○	○	○	○
Reorganizing roles among family members	○	○	○	○	○
Taking care of bills	○	○	○	○	○
Talking with other people about your deceased family member's illness	○	○	○	○	○
Dealing with emotional distress of other family members (e.g., anger, anxiety, depression, fear, resentment, etc.)	○	○	○	○	○
Finding meaning out of your experience with your deceased family member's illness	○	○	○	○	○
Getting help with your household activities	○	○	○	○	○

Understanding medical or insurance coverage					
Being satisfied with your relationship with other family members and friends					
Taking care of your own health					
Dealing with lifestyle changes					
Dealing with emotional distress (e.g., anger, anxiety, depression, fear, resentment, etc.)					
Getting help from others in order to take time for yourself					
Meeting your personal needs					
Getting together with family and friends					
Taking time off from work					
Understanding or navigating the health care system					
Having an intimate relationship					

Helping other family members find meaning out of your deceased family member's illness	○	○	○	○	○
Getting legal paperwork done	○	○	○	○	○

Display This Question:

If Next, we will ask you several questions about your needs related to DC or a related TBD. Based on... = a bereaved parent or legal guardian of a child who had DC or a related TBD

And And During the past 4 weeks, have you experienced a need related to any of the following? Mark all... q://QID57/SelectedChoicesCount Is Greater Than or Equal to 1

Carry Forward Selected Choices from "During the past 4 weeks, have you experienced a need related to any of the following? Mark all that apply."

During the past 4 weeks, how satisfied have you been with fulfilling the need to deal with...

	Not at all	A little bit	Somewhat	Quite a bit	Extremely
Taking part in your usual social or recreational activities	○	○	○	○	○
Talking to others who have lost a loved one to the same illness	○	○	○	○	○
Reorganizing roles among family members	○	○	○	○	○
Taking care of bills	○	○	○	○	○
Talking with other people about your deceased family member's illness	○	○	○	○	○
Dealing with emotional distress of other family members (e.g., anger, anxiety, depression, fear, resentment, etc.)	○	○	○	○	○
Finding meaning out of your experience with your deceased family member's illness	○	○	○	○	○
Getting help with your household activities	○	○	○	○	○

Understanding medical or insurance coverage	○	○	○	○	○
Being satisfied with your relationship with other family members and friends	○	○	○	○	○
Taking care of your own health	○	○	○	○	○
Dealing with lifestyle changes	○	○	○	○	○
Dealing with emotional distress (e.g., anger, anxiety, depression, fear, resentment, etc.)	○	○	○	○	○
Getting help from others in order to take time for yourself	○	○	○	○	○
Meeting your personal needs	○	○	○	○	○
Getting together with family and friends	○	○	○	○	○
Taking time off from work	○	○	○	○	○
Understanding or navigating the health care system	○	○	○	○	○
Having an intimate relationship	○	○	○	○	○

Helping other family members find meaning out of your deceased family member's illness	◯	◯	◯	◯	◯
Getting legal paperwork done	◯	◯	◯	◯	◯
