# Supplementary material for: Defining the complex needs of families with rare diseases—the example of telomere biology disorders
Source: Eur J Hum Genet. 2024 Oct 1;32(12):1615–23. doi: 10.1038/s41431-024-01697-6 (PMC11607413; doi:10.1038/s41431-024-01697-6)
Supplement: Supplementary file 3 — Supplementary Material 3 [file 41431_2024_1697_MOESM3_ESM.pdf]

Supplementary Material 3: Researcher positionality and reflexivity

Who we are: managing and acknowledging team identities

Our large interprofessional team was actively involved in data collection and analysis. Our team values were positioned within the traditions of applied, person-centered research: we non-judgmentally explored experiences of disease to inform future research that aims to improve patient, family, and community-defined outcomes. Participant perspectives were treated as expert accounts of living with a telomere biology disorder (TBD) as a patient and/or caregiver and assumed to represent participant realities (*i.e.*, experiential orientation). We intentionally made use of different disciplinary angles and interpretations to allow for rich and in-depth analysis of participant stories. Represented disciplines included: social work, medicine, nursing, genetic counseling, dietetics, behavioral science, and public health. Three team members were actively involved in the clinical care of patients and families with TBDs, CW (social work), AST (genetic counseling), and SAS (medicine). There was a range of qualitative research experience on our interview and coding team, with three experts (RFS, CW, CJR; collectively conducting >200 interviews among cancer/familial cancer populations in different settings), and team members in training for qualitative interviewing and analysis (EEP, AST, AM). Six team members identified as cis-gender female, one as male.

We managed our positions and identities in the research process with an integrated, team-based approach, meeting regularly in multi-disciplinary team meetings. No single discipline predominated; perspectives and insights were encouraged from all team members, regardless of experience. To track team-member assumptions, positionality, and tensions within data, coders maintained a memo writing practice during data collection and analysis, using reflexive prompts adapted from Sunstein and Chiseri-Strater (2007) (*e.g.*, What surprised, intrigued, or unsettled me?) Key examples of these are provided below.

Assumptions: TBDs are inherited, but distinctive; be wary of foreclosure

Having developed expertise working with high-risk genetic populations, we had to be mindful to preserve an openness and curiosity to specific TBD experiences without foreclosing on them because they 'sound similar' to reports from other work. By creating an expansive codebook and sharing the analytical workload across the team, we were able to preserve the breadth of TBD experiences while also freeing up team members to deeply analyze and conceptualize sections of the codebook for which they had disciplinary expertise (*e.g.*, discipline: social work; codebook domain: psychosocial needs and discipline: nursing; codebook domain: medical needs).

Positionality: we do not have lived TBD experience and be ready to 'be there' for participants

Our team did not possess first-hand experience of living with a TBD as a patient or caregiver. We actively and intentionally engaged Team Telomere to guide our enquiry from inception to publication, seeking feedback from Team Telomere leaders at key intervals (*e.g.*, development of research questions, interview guide, and candidate themes). From experience with other rare disease groups, we anticipated that the interview process could be therapeutic for many people as they have the opportunity to share, with a ready and curious listener, life experiences often kept private. To ensure interviewers could maintain active and empathetic engagement with the experiences of participants (*e.g.*, grief, loss, and suffering), we practiced debriefing to explore

vicarious feelings of discomfort and coordinated a larger data collection team (five interviewers) to spread interview load.

Tensions: identifying unmet needs without being able to intervene

As a team, we recognized the limitations of our descriptive qualitative analysis. Coming from interpretive and clinical disciplines, we felt a responsibility to intervene (*e.g.*, provide mental health counseling) that could not be fulfilled in the current study. To address this tension, we have and will continue to disseminate our findings in relevant clinical settings and at scientific and TBD community events to bring attention to the numerous gaps in meeting the needs of individuals and families with TBDs. We documented all analytical insights pertaining to maximizing our qualitative dataset to illuminate the extent of unmet needs in the TBD population, including the possibility of conducting additional in-depth analyses and using findings to inform the development of future intervention research.

References:

Sunstein, B. S., & Chiseri-Strater, E. (2007). *Fieldworking: Reading and writing research (3rd ed.)*. Bedford/St Martin’s Press.
